# Quantifying Ecological Integrity of Terrestrial Systems to Inform Management of Multiple-Use Public Lands in the United States

**DOI:** 10.1007/s00267-019-01163-w

**Published:** 2019-04-13

**Authors:** Sarah K. Carter, Erica Fleishman, Ian I. F. Leinwand, Curtis H. Flather, Natasha B. Carr, Frank A. Fogarty, Matthias Leu, Barry R. Noon, Martha E. Wohlfeil, David J. A. Wood

**Affiliations:** 1 U.S. Geological Survey, Fort Collins Science Center, 2150 Centre Ave. Building C, Fort Collins, CO 80526-8118 USA; 2 Department of Fish, Wildlife and Conservation Biology, Colorado State University, Fort Collins, CO 80523 USA; 3 Cherokee Nation Technologies, on contract to U.S. Geological Survey, Fort Collins Science Center, Fort Collins, CO 80526 USA; 4 U.S. Department of Agriculture Forest Service, Rocky Mountain Research Station, Fort Collins, CO 80526 USA; 5 Department of Environmental Science and Policy, University of California - Davis, Davis, CA 95616 USA; 6 Biology Department, College of William and Mary, Williamsburg, VA 23187 USA; 7 Bureau of Land Management, National Operations Center, Denver, CO 80255 USA; 8 Present Address: Conservation Science Partners, 5 Old Town Square, Suite 205, Fort Collins, CO 80524 USA; 9 Present Address: U.S. Geological Survey Northern Rocky Mountain Science Center, 2327 University Avenue, Suite 2, Bozeman, MT 59715 USA

**Keywords:** Bureau of Land Management, Nevada, Shrublands, Reference conditions, Ecosystem services, Land health

## Abstract

The concept of ecological integrity has been applied widely to management of aquatic systems, but still is considered by many to be too vague and difficult to quantify to be useful for managing terrestrial systems, particularly across broad areas. Extensive public lands in the western United States are managed for diverse uses such as timber harvest, livestock grazing, energy development, and wildlife conservation, some of which may degrade ecological integrity. We propose a method for assessing ecological integrity on multiple-use lands that identifies the components of integrity and levels in the ecological hierarchy where the assessment will focus, and considers existing policies and management objectives. Both natural reference and societally desired environmental conditions are relevant comparison points. We applied the method to evaluate the ecological integrity of shrublands in Nevada, yielding an assessment based on six indicators of ecosystem structure, function, and composition, including resource- and stressor-based indicators measured at multiple scales. Results varied spatially and among indicators. Invasive plant cover and surface development were highest in shrublands in northwest and southeast Nevada. Departure from reference conditions of shrubland area, composition, patch size, and connectivity was highest in central and northern Nevada. Results may inform efforts to control invasive species and restore shrublands on federal lands in Nevada. We suggest that ecological integrity assessments for multiple-use lands be grounded in existing policies and monitoring programs, incorporate resource- and stressor-based metrics, rely on publicly available data collected at multiple spatial scales, and quantify both natural reference and societally desired resource conditions.

## Introduction

There is a strong and growing focus on managing natural systems for holistic objectives such as ecological integrity (e.g., Hobbs et al. 2010; Reza and Abdullah 2011; USFS 2015). The concept of ecological integrity emerged in the 1940s (Leopold 1949) and became a legally mandated objective for aquatic systems in the United States upon enactment of the Clean Water Act of 1972 (33 USC §1251). Ecological integrity has been adopted as an objective for aquatic systems by the European Union and other jurisdictions worldwide (e.g., European Union Water Framework Directive [2000/60/EC], Borja et al. 2008). As a result, methods to quantify ecological integrity in different aquatic systems and at different spatial scales have been developed and refined (Karr et al. 1987; Fayram et al. 2005; Borja et al. 2008, 2009; Reza and Abdullah 2011; Wilson and Bayley 2012; Ruaro and Gubiani 2013). Programs and regulations designed to preserve and restore ecological integrity have improved the status of many impaired water bodies (USGAO 2013).

**Fig. 1:**
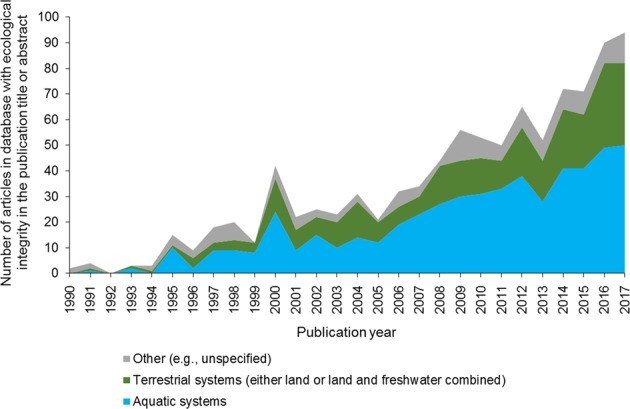
The number of articles published in the peer-reviewed literature on ecological integrity that focused on aquatic systems, terrestrial systems (including land or land and freshwater combined), or other (e.g., unspecified) from 1990 to 2017. Search conducted March 2018 in Web of Science (webofknowledge.com) using a topical search for the term “ecological integrity” and excluding articles that did not include the search term in the article title or abstract

Regulations establishing ecological integrity as an objective for terrestrial systems are less prevalent and also, with the exception of the Wilderness Act of 1964 (16 U.S.C. §§ 1131–1136), have been adopted more recently than those for aquatic systems (e.g., Refuge Improvement Act of 1997, Canada National Parks Act [S.C. 2000, c. 32], U.S. Forest Service Planning Rule (36 CFR 219 [2012])). Far fewer publications have addressed the ecological integrity of terrestrial systems than aquatic systems (Fig. 1). Furthermore, the application of ecological integrity to multiple-use resource management contexts, in which some resource objectives may directly conflict with conservation goals, is not well developed (Wurtzebach and Schultz 2016).

Multiple use refers to management of public lands and their resource values to best meet present and future societal needs (e.g., USDOI 2001). Multiple-use policies apply to lands under the jurisdiction of many federal entities in the United States, including the Bureau of Land Management (BLM; Federal Land Policy and Management Act of 1976 [43 USC §1701]), Forest Service (Multiple-Use Sustained-Yield Act of 1960 [16 USC §528]), and Department of Defense (USDOD 2016). Common resource uses on multiple-use lands include livestock grazing, timber harvest, energy production, mineral extraction, recreation, and harvest of fishes and terrestrial animals. Laws and policies applicable to multiple-use lands also require protection of the “quality of scientific, scenic, historical, ecological, environmental, air and atmospheric, water resource, and archeological values” of these lands (Federal Land Policy and Management Act of 1976); maintaining or restoring land health (Fundamentals of Rangeland Health [43 CFR §4180.1]); and maintaining or restoring ecological integrity and ecosystem diversity (U.S. Forest Service 2012 Planning Rule [36 CFR §219.9(a)]). Indeed, the objectives for many public and protected lands worldwide are accommodation of recreational and extractive uses that support local economies while conserving natural resources (Naughton-Treves et al. 2005).

The BLM manages more land in the United States than any other federal agency or department (1,004,358 km^2^, USDOI 2016), and is charged with accommodating sustainable development of energy and minerals on the lands within its jurisdiction (USDOI 2017) while protecting the health of those lands and watersheds (Fundamentals of Rangeland Health [43 CFR §4180.1]). The term *ecosystem health* is commonly used, but rarely defined clearly (O’Brien et al. 2016). Most definitions (Rapport et al. 1998) and assessments (e.g., Edson et al. 2016; Hansen and Phillips 2018) focus on the current functioning of the ecosystem and its connections to people through the provision of ecosystem services. Ecological integrity, a related concept, has emerged as a holistic foundation for management of public parks and wilderness areas (Hobbs et al. 2010). Unlike most other lands for which ecological integrity assessments have been conducted (e.g., individual national parks), BLM lands are extensive, often disjunct (e.g., the wide swaths of checkerboard BLM jurisdiction across northern Nevada and southern Wyoming), and must accommodate resource extraction and conservation objectives that can change rapidly and unpredictably (BLM 2015a, 2017c).

To help inform its management actions, BLM recently implemented a standardized monitoring program across the western United States to streamline collection of natural resource data (Toevs et al. 2011). As a result, BLM sought to operationalize the concept of ecological integrity, relying primarily on these and other existing data. Accordingly, our objectives here were to develop a transferable method for quantifying ecological integrity across extensive, multiple-use terrestrial landscapes in the western United States, and to apply that method to public lands managed by the BLM in Nevada. Some concepts in this article were presented in an earlier U.S. Geological Survey report (Carter et al. 2017). This article expands on and operationalizes those concepts, includes considerable material not presented in the earlier report, and adds a quantitative case study.

### Defining Ecological Integrity

We define ecological integrity as the extent to which the composition, structure, and function of an ecosystem fall within their natural range of variation. This definition builds on earlier work (Table 1). Natural range of variation refers to the values of a metric likely to be observed under natural reference conditions (i.e., in the absence of human disturbance [Stoddard et al. 2006]). Given the rarity of natural reference conditions available today (e.g., Vitousek et al. 1997; Sanderson et al. 2002; Leu et al. 2008; Theobald 2013), the natural range of variation often is applied in practice as a probability distribution of conditions likely to have occurred during a defined period in history (e.g., the period prior to EuroAmerican settlement commonly is referenced in Canada and the United States, which broadly equates to the 19th or early 20th century in the western United States). Composition (the identity and variety of elements), structure (physical organization or pattern), and function (ecological and evolutionary processes) are the primary attributes of ecosystems (Franklin et al. 1981) and other levels of biological organization (Wilcox 1984;, Noss 1990), and encompass other terms often included in the definition of ecological integrity (e.g., diversity). We suggest that the concepts of resistance, resilience, and recovery referenced in some definitions of ecological integrity (Table 1) be considered as potential metrics of ecosystem function.

**Table 1:** Illustrative definitions of ecological and biotic integrity

Source	Primary context	Definition
Karr and Dudley 1981	Regulation of freshwater systems through the U.S. Clean Water Act	“The capability of supporting and maintaining a balanced, integrated, adaptive community of organisms having a species composition, diversity, and functional organization comparable to that of natural habitat of the region.”
		“A system possessing integrity can withstand, and recover from, most perturbations imposed by natural environmental processes, as well as many major disruptions induced by man.”
Canada National Parks Act (S.C. 2000, c. 32, s. 2)	Management of national parks	“A condition that is determined to be characteristic of its natural region and likely to persist, including abiotic components and the composition and abundance of native species and biological communities, rates of change and supporting processes.”
Miller 2000; Ulanowicz 2000	Integration of environment, conservation, and health in a sustainable development context, shared mutual self-interest, and biophilia	Four key attributes: “(1) System health … the continued successful functioning of the community, (2) the capacity to withstand stress, (3) an undiminished ‘optimum capacity’ for the greatest possible ongoing development options, and (4) the continued ability for ongoing change and development, unconstrained by human interruptions.”
Parrish et al. 2003	Management of protected areas	“The ability of an ecological system to support and maintain a community of organisms that has species composition, diversity, and functional organization comparable to those of natural habitats within a region.” “The dominant ecological characteristics of a system or species … can withstand and recover from most perturbations imposed by natural environmental dynamics or human disruptions.”
This paper	Management of multiple-use lands	The extent to which the composition, structure, and function of an ecosystem fall within their natural range of variation.

Natural reference conditions and the associated natural range of variability are the benchmark for assessing the ecological integrity of multiple-use systems. Societally desired conditions are a second relevant comparison point. Desired conditions reflect societal goals for the system, including desired resource uses and ecosystem goods and services, in light of its current social and landscape context. Desired conditions often exist along a gradient, the bounds of which represent a social range of variability (Duncan et al. 2010). Society may identify desired conditions via laws, policies, or plans. For example, federal regulations may require maintenance of a minimum water quality standard or a minimum area of habitat for an endangered species, and states may identify maximum allowable areas of surface disturbance in habitat for a given species (e.g., 5% surface disturbance caps in areas of priority habitat for Greater Sage-Grouse [*Centrocercus urophasianus*] in Wyoming; State of Wyoming 2011). Societally desired conditions provide important context for understanding how historic conditions and the current status of the system compare with current management objectives.

Desired conditions may fall within or outside the natural range of variation. Areas designated as strict nature reserves (IUCN category Ia) are managed to protect biological diversity and geological features and to retain intact ecosystems (Dudley 2008). Thus, the desired conditions for a strict nature reserve overlap with the natural range of variation. By contrast, to meet societal needs and desires for energy, agriculture, transportation, and housing, society has reduced the frequency and extent of flooding on many large rivers to levels outside natural ranges of variation. The extent of overlap between natural reference and societally desired conditions varies in time and space in response to social, legal, economic, and political factors. Decreases in system integrity become more likely as natural reference and societally desired conditions diverge (Fischer and Lindenmayer 2007, Duncan et al. 2010).

### Ecological Integrity and Multiple-Use Lands

Multiple-use lands fall along a continuum, from largely intact areas for which most ecological metrics are within their natural range of variation, to highly modified lands for which most ecological metrics are well outside of their natural range of variation. Zones along this continuum can be characterized as protective, compromise, or productive (Karr and Dudley 1981), with an associated change in the major types of ecosystem services provided (Wu 2013). Strategies for allocating different portions of landscapes to different zones along this continuum have been characterized as land sparing versus land sharing (Phalan et al. 2011; Fischer et al. 2014). Protected areas, such as designated wilderness areas in the United States, are largely intact and thus fall toward the protective end of the continuum, but still may be affected by past land uses (e.g., use of fire by indigenous peoples or deforestation in the early 1800s), current land uses (e.g., recreation), ecological changes (e.g., colonization by non-native invasive species), and landscape context (e.g., land uses in the surrounding area). National parks generally have lower integrity than wilderness areas because they typically are smaller, exacerbating edge effects (Radeloff et al. 2010; Gimmi et al. 2011), and have a higher density of roads and infrastructure to accommodate diverse recreational uses. Many multiple-use public lands managed by the BLM are compromise lands that accommodate activities, such as drilling for oil and gas, which may negatively affect metrics of ecological integrity (Jones et al. 2015). However, in certain ecological circumstances, some resource extraction activities can increase ecological integrity. For example, silvicultural treatments, which often are implemented to provide commercial timber to local communities, also can reduce fuel loads and restore desirable vegetation structure (Littell et al. 2009; Spies et al. 2014). Ecological integrity assessments can provide valuable information on the extent to which past management may have decreased the integrity of multiple-use lands, and repeated assessments can help to evaluate the effectiveness of current management actions. Some productive lands such as agricultural fields may retain high ecological and conservation value when managed at lower intensities as part of heterogeneous landscapes (e.g., Bignal and McCracken 1996; Dooresteijn et al. 2015). We suggest that assessments of ecological integrity are most useful for protective and compromise lands, although integrity assessments also have been conducted for productive lands (Stoll et al. 2015).

## A Method for Quantifying Ecological Integrity to Inform Multiple-Use Land Management

Although the concept of ecological integrity is intuitive and appealing, it is difficult to quantify. There is no single metric, taxonomic group, or scale at which integrity can be quantified for all ecosystems (e.g., Diffendorfer et al. 2007; Lindenmayer et al. 2015; but see Ulanowicz 2000, 2004). The natural and anthropogenic drivers of a system, and stressors to integrity (e.g., point-source pollution, climate change), operate and interact at different extents (e.g., Urban et al. 1987; Bestelmeyer et al. 2003; Seidl et al. 2016). Different attributes of natural systems (e.g., a species’ abundance, genetic diversity, or movement behavior) may respond to a given disturbance in different ways and at different scales (Jackson and Fahrig 2012, 2014). Ecosystem services also are provided, interact, and respond to management at different spatial and temporal scales (Costanza 2008; Stoll et al. 2015; Lindborg et al. 2017). Some ecological integrity assessments may require extensive sampling (e.g., Patricio et al. 2006) that is not feasible for many management agencies. Current assessment methods also may not identify likely causes of changes in ecological integrity (King 1993), making it difficult to understand which management actions are most likely to improve ecological integrity.

**Fig. 2:**
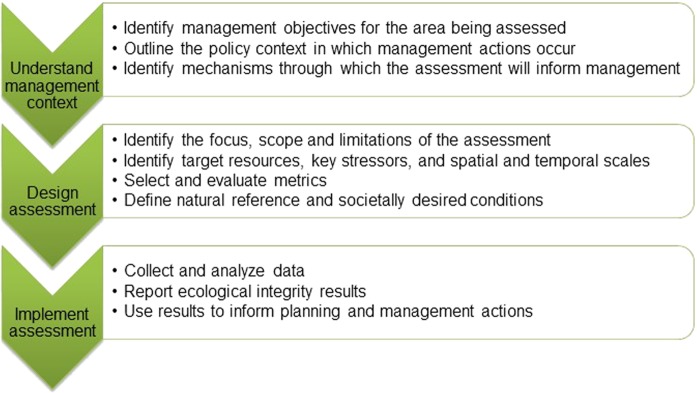
Stages (arrows) and steps (bulleted) in assessing ecological integrity to inform management of multiple-use lands

Building on previous work (Parrish et al. 2003; Tierney et al. 2009; Mitchell et al. 2014), we outline a three-stage method to design and conduct ecological integrity assessments for large, multiple-use systems that aims to address these challenges and the need to operationalize the integrity concept for informing multiple-use land management (Timberlake and Schultz 2017) (Fig. 2). The stages explicitly link the assessment to management policies and actions, clarify which resources and stressors can be managed, and define both natural reference and societally desired conditions. The method and steps are purposely flexible to allow the assessment to be tailored to different landscape and management contexts. Although we illustrate the method as linear for simplicity, the steps are likely to be iterative and to have multiple feedback loops.

### Understanding the Management Context

The first stage of the assessment is characterization of the management context—the laws, policies, and plans that guide management actions in the area. Less than one-quarter of ecological integrity assessments in freshwater systems are related to a specific freshwater policy (Kuehne et al. 2017). The foundation for a practical assessment that resource managers readily can use lies in understanding the major laws and policies that currently guide resource management in the area (e.g., requirements related to the Fundamentals of Rangeland Health [43 CFR §4180.1], impaired waters [303(d) of the Clean Water Act of 1972, 40 CFR §130.7], or endangered species [Section 7 of the Endangered Species Act of 1973 as amended, 16 USC. §1536]). Identifying current and emerging management concerns and objectives for the assessment area (from property or land-use plans and the scientific literature) and mechanisms through which the assessment may inform management actions (e.g., desired conditions and trigger points for action in property or land-use plans) also increases the likelihood that the assessment will be useful for and used by managers.

### Designing the Assessment

**Fig. 3:**
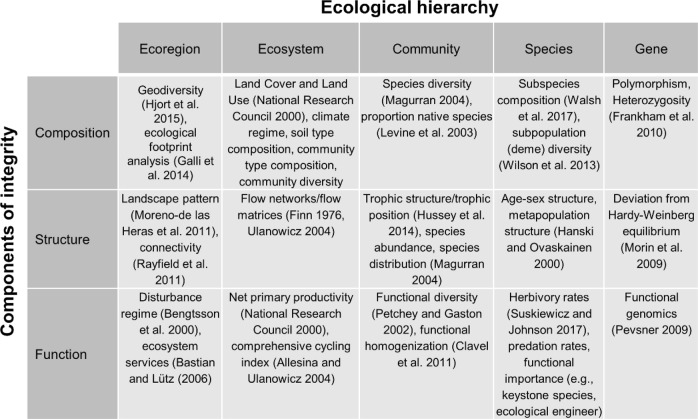
A conceptual framework for ecological integrity assessments and example metrics

The second stage is designing the assessment in light of the management context. We conceptualized ecological integrity assessments along two dimensions. The first represents components of integrity (composition, structure, and function) and the second represents levels in the ecological hierarchy (ecoregion, ecosystem, community, species, and gene, Fig. 3). Given that multiple metrics likely are necessary to characterize each element, and ranges of variation must be quantified for each metric, comprehensive assessments rarely will be possible. Accordingly, the main purpose of this framework is to facilitate identification of focal elements for a given assessment, which then characterizes both the scope and the limitations of the assessment.

Selection of focal elements will be informed by the ecology of the system and by its management, social, and economic context. Assessments of ecological integrity on multiple-use lands likely will quantify the status of priority resources and stressors over which there is management control (e.g., livestock grazing or energy production infrastructure) or growing management concern (e.g., climate change). Resource uses that may degrade ecological systems (e.g., energy and mineral development, grazing by domestic livestock, timber harvest) are those most often managed or restricted by land-use plans.

We suggest that natural and anthropogenic processes structuring the system be identified in a standardized manner (Salafsky et al. 2008), and their hypothesized effects on target resources identified in a conceptual model (Ogden et al. 2005; Lindenmayer et al. 2008). Considering the effects of unmanaged processes on target resources increases the likelihood of accurately evaluating integrity metrics against the natural or desired ranges of variation and predicting the metric’s response to management actions (Irvine et al. 2015).

We suggest that the spatial and temporal scales of the assessment reflect the natural and anthropogenic drivers of the system and be informed by its social and management context (Vogt et al. 2002). Resource stressors may operate, for example, at local levels (e.g., an oil or gas well pad) or regional levels (e.g., changes in fire regimes across the southwestern United States). Management actions may be applied at individual sites (e.g., spot weed treatments), across multiple properties managed by an agency (e.g., Greater Sage-Grouse priority habitat management areas), or across jurisdictions (e.g., collaborative restoration initiatives such as the Wyoming Landscape Conservation Initiative; Bowen et al. 2018). Stakeholders may be geographically concentrated or dispersed. Temporal scales for the assessment may be informed by biological considerations (e.g., time lags in the response of resources to perturbations, recovery time following disturbances; Lindenmayer et al. 2008), management constraints (e.g., allowable reference periods), and planning and permitting time frames. As a result, most comprehensive assessments will consider information collected at multiple scales (Andreasen et al. 2001; Lindenmayer et al. 2008; Wurtzebach and Schultz 2016) and ecological levels (O’Brien et al. 2016). Boundaries of an assessment may be ecological (e.g., watersheds), institutional (e.g., park borders), or political (e.g., states). Assessments are likely to be used only if their extent encompasses the administrative unit responsible for the planning or management action (e.g., BLM field office, National Forest), but it may be helpful or necessary to expand the boundary beyond the administrative unit (e.g., intersecting watersheds) to accurately quantify some ecological integrity metrics.

Metrics of ecological integrity ideally are relevant to and interpretable by all stakeholders, feasible to measure and monitor consistently over the long term, amenable to statistical analysis and change detection, and responsive to stressors and management actions (e.g., Andreasen et al. 2001; Kurtz et al. 2001; Noon 2003; Reza and Abdullah 2011; Game et al. 2018). Use of metrics that are responsive to management, together with information on the application of management actions, also can facilitate evaluations of management effectiveness (Tierney et al. 2009; Timko and Innes 2009; McDonald-Madden et al. 2010). Ideally, each metric will provide complementary information on structure, composition, or function of the system (Brown and Williams 2016; O’Brien et al. 2016; Kuehne et al. 2017). Metrics of ecosystem function have not been emphasized in previous assessments (e.g., Timko and Innes 2009). However, Meyer et al. (2015) proposed metrics of ecosystem function that are feasible to measure repeatedly over time and in different systems.

Numerous assessments have used community-level metrics of macroinvertebrates, fishes, or birds to quantify ecological integrity (e.g., O’Connell 2009; O’Brien et al. 2016). Although federal land management agencies in the United States monitor wildlife directly in some cases (e.g., US Fish and Wildlife Service 2007, Lint et al. 1999), they often focus primarily on monitoring and managing vegetation, water, and soil (e.g., Toevs et al. 2011, but see Nie et al. 2017). Use of discrete measures of the resources that agencies regularly manage and monitor in the area of interest provides a clear connection to the laws, policies, and plans that guide their work.

Inclusion of stressor-based metrics in integrity assessments is becoming more common (Kuehne et al. 2017; McGarigal et al. 2018). These metrics help to focus assessments on current and emerging threats (e.g., Cleland et al. 2017), potentially increasing the efficiency of conservation actions (e.g., Newburn et al. 2005; Bottrill et al. 2009). Although some ecological integrity assessments are based solely on stressor-based metrics (e.g., Theobald 2013; Decker et al. 2017), we suggest that assessments include both resource- and stressor-based metrics so that there are direct connections both to the resources the agency is responsible for managing and the resource uses that the agency permits. Stressor-based metrics also can provide a direct link to decision making through adaptive management thresholds (Nie and Schultz 2011).

Clearly defining and justifying the natural range of variation in each ecological integrity metric makes explicit the benchmarks to which current values of each metric will be compared. Established alternatives to true reference condition include minimally disturbed condition (the condition of systems in the absence of substantial human disturbance) and historical condition (the condition of systems at a specified point in their history, often pre-EuroAmerican settlement, Stoddard et al. 2006; Binkley and Duncan 2009). However, not all values within the natural or historic range of variability of a system may be acceptable given current land uses (e.g., stand-replacing fires in the wildland-urban interface, major floods in agricultural areas).

Desired conditions may reflect what is believed to be the best attainable condition of the system given its current regulatory and landscape context (Stoddard et al. 2006), the conditions necessary to ensure long-term sustainability of the system and persistence of target resources (Andreasen et al. 2001; Parrish et al. 2003), or other states that provide the mix of ecosystem services currently desired by society in light of historical, ecological, economic, and social considerations (Dombeck 1996). As such, desired conditions are a second relevant comparison point for ecological integrity metrics. However, ecological integrity metrics that fall within the range of desired conditions do not imply that the system has integrity. Instead, desired conditions provide context for understanding the distance between current conditions, current management objectives, and true reference conditions. Desired conditions often are defined in land-use or management plans for individual species or protected areas (e.g., Commissioner of the Environment and Sustainable Development 2013). For example, BLM’s land-use plan for the California desert limits surface disturbance to 1% of modeled suitable habitat for a suite of sensitive plant species (BLM 2016). When natural reference or societally desired conditions are unknown or unspecified, quantile scaling provides a mechanism for comparing relative levels of ecological integrity among sites (McGarigal et al. 2018).

### Implementing the Assessment

The third stage is to conduct the assessment. Given limited budgets, existing agency monitoring programs ideally will provide the data needed for integrity assessments. Most monitoring within the BLM is conducted through the agency’s Assessment, Inventory, and Monitoring (AIM) program (Toevs et al. 2011). Standardized data collected through the AIM program are intended to meet multiple agency monitoring and assessment needs at multiple spatial scales. Integration of field-collected and remotely sensed metrics is a major goal of the AIM program (Toevs et al. 2011), and allows for validation of remotely sensed metrics. Use of metrics for which data are publicly accessible promotes transparency and accountability and facilitates a shared understanding of resource conditions and uses, all of which can increase stakeholder involvement in management and lead to better long-term management outcomes (e.g., Sayer et al. 2013).

Ideally, decisions about how to communicate results of an ecological integrity assessment should be made in consultation with all parties with an interest in management of the system. A number of visual, user-friendly formats are available (e.g., Cardoso et al. 2007; Tierney et al. 2009; Mitchell et al. 2014). Use of a composite index of ecological integrity metrics may facilitate communication with policy makers and the public (Wurtzebach and Schultz 2016), but results in loss of information, decreases the ability to explore factors that may be driving changes in integrity, and is statistically problematic (Brown and Williams 2016). In addition, variation in the direction and magnitude of responses of individual metrics to stressors can be masked in a composite index (Norris and Hawkins 2000). Presenting the status of metrics individually is most informative for managers (Mitchell et al. 2014; Brown and Williams 2016; Wurtzebach and Schultz 2016). For example, the species composition of grasses is related to the intensity of grazing by domestic livestock (DiTomaso 2000), potentially informing future decisions about livestock stocking rates. Therefore, we suggest that metrics not be combined. Although results for each metric may be presented categorically for simplicity, retaining the underlying continuous data is essential to avoid loss of information (Brown and Williams 2016).

The ultimate aim of ecological integrity assessments is to inform future resource planning and management actions, which may include more intensive monitoring of actions that may be causing a departure from reference or desired conditions. Commonly suggested approaches for understanding and managing complex systems include active adaptive management, scenario planning, maximizing sustained yield, and building resilience (Allen and Gunderson 2011). Decision support methods such as structured decision making can provide guidance and tools for implementing each (Schwartz et al. 2017). Iterative assessments can be used to evaluate both the hypotheses underlying the assessment and the effectiveness of management actions intended to increase ecological integrity (Bottrill and Pressey 2012; Mitchell et al. 2014; Irvine et al. 2015).

## Case Study: Assessing the Ecological Integrity of Shrublands in Nevada

### Understanding the Management Context

**Fig. 4:**
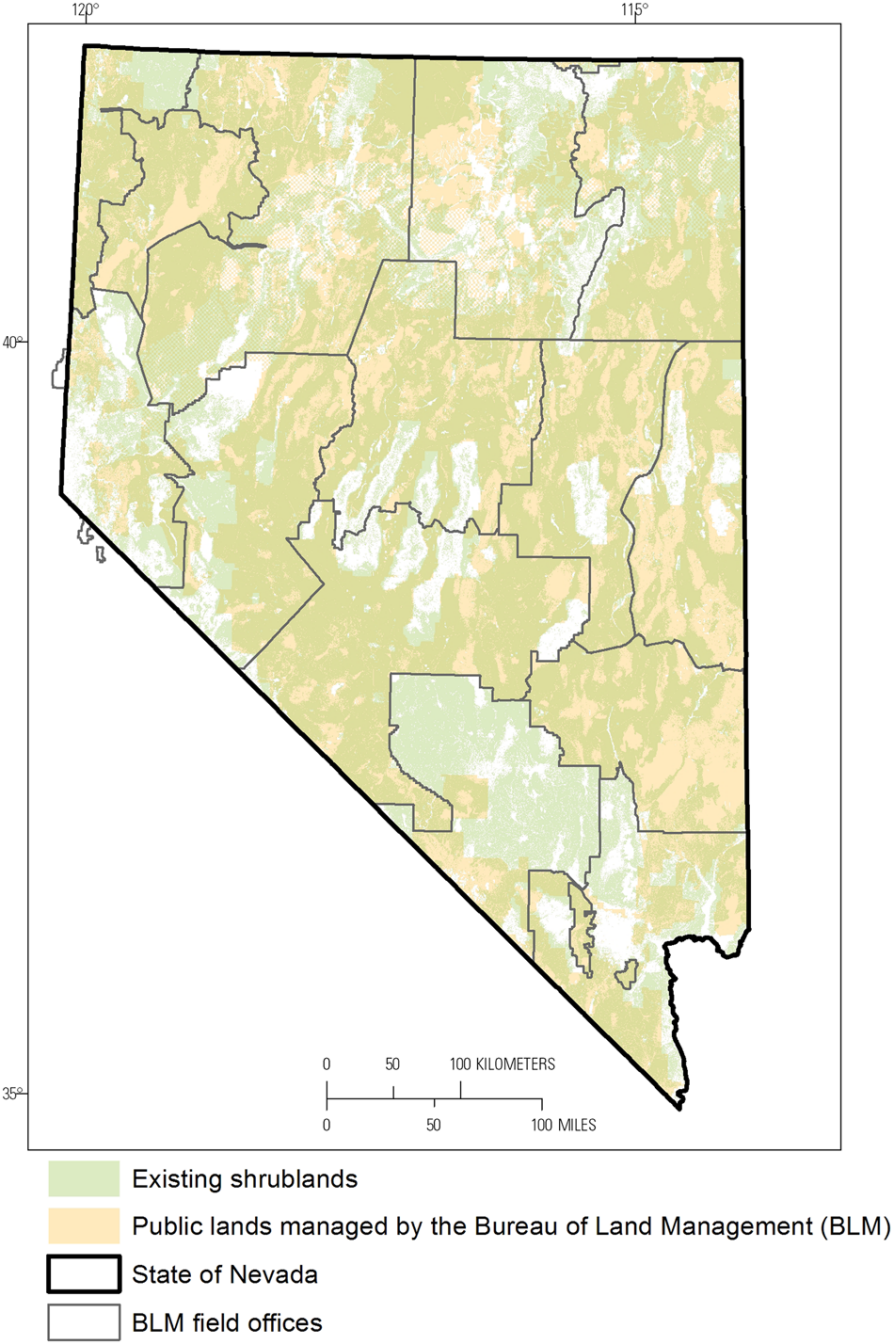
Current and historic shrublands and public lands managed by the Bureau of Land Management (BLM) in Nevada. Fourteen BLM field offices are contained entirely within Nevada and were considered for this study. See Fig. 6 for the names and abbreviations of BLM field offices

We applied the method outlined above to assess the ecological integrity of shrublands in Nevada. Shrublands covered most of Nevada in the centuries before westward expansion, and the majority of contemporary shrublands are on public lands managed by the BLM (Fig. 4). The management context for the assessment is framed by the BLM’s mission to manage public lands for multiple resource uses and values (Federal Land Policy and Management Act of 1976) and to maintain or restore watershed health, ecological processes, water quality, and habitat for special status species (43 CFR §4180.1). BLM resource management plans, generally conducted at the level of individual field offices (the majority of the area of 14 BLM field offices is in Nevada; Fig. 4), identify objectives and desired conditions for the lands managed by BLM within the planning boundary. For example, the vegetation objective in an eastern Nevada resource management plan is to “manage for resistant and resilient ecological conditions including healthy, productive, and diverse populations of native and desirable non-native plant species appropriate to the site characteristics” (BLM 2008). Some shrublands in Nevada provide habitat for Greater Sage-Grouse, a species of major conservation concern across much of the western United States (USFWS 2015). Maintaining and restoring the integrity of these shrublands is a high priority for BLM (BLM 2015a).

### Designing the Assessment

We focused the assessment at the ecosystem level (see Fig. 3) to support BLM’s commitment to ecosystem management (Dombeck 1996) and used the 14 BLM field offices in Nevada as our spatial analysis units because field office boundaries generally correspond with the boundaries of BLM land-use plans (called resource management plans). BLM is required to monitor and evaluate progress toward achieving objectives in land-use plans at least every 5 years (43 CFR §1610.4–9, BLM 2015b).

We used conceptual models of Nevada ecosystems (Comer et al. 2013) and information on metrics currently monitored (Taylor et al. 2014) or being considered for monitoring (Wood et al. 2017) across the western United States by the BLM’s AIM program to identify metrics of the structure, function, and composition of shrublands in Nevada (Table 2). The physiography of the region is characterized by many long, narrow mountain ranges separated by broad valleys, and a semiarid climate with large daily and seasonal temperature fluctuations. Other major natural drivers of dryland systems in the region include infiltration of soil moisture, erosion, accumulation of soil organic matter, and natural disturbance (Comer et al. 2013). Major stressors to shrublands in Nevada, which operate at temporal scales from years to decades and are closely related, are changes in wildfire extent, intensity, and frequency; expansion of cheatgrass (*Bromus tectorum*), a non-native, invasive, and highly flammable annual grass; and grazing by domestic livestock and wild horses and burros (Milchunas 2006; Comer et al. 2013; Dalldorf et al. 2013). Although the density of human settlement in Nevada generally is low, land conversion, recreation, hunting, and conflicts between humans and animals can stress resources, and the number, density, and size of renewable energy projects is becoming a management concern (Comer et al. 2013).

**Table 2:** Metrics for quantifying the compositional, structural, and functional components of ecological integrity of shrublands in Nevada, the spatial scale (local or broad) at which the metrics are assessed and quantitative reference conditions available for this study

Metric	Composition	Structure	Function	Data and source	Reference condition and source
Vegetation composition	Local			(BLM 2017a)	–
Presence of plants of management concern	Local			(BLM 2017a)	–
Percent cover of non-native invasive plants	Local			(BLM 2017a)	Absence of non-native invasive species
Vegetation height		Local	Local	(BLM 2017a)	–
Percent cover of bare ground		Local	Local	(BLM 2017a)	–
Proportion of soil surface in large canopy gaps		Local	Local	(BLM 2017a)	–
Vegetation area	Broad			Existing vegetation (LANDFIRE 2014a)	Biophysical setting (LANDFIRE 2010)
Vegetation alteration	Broad	Broad	Broad	Vegetation departure (LANDFIRE 2014b)	Biophysical detting (LANDFIRE 2010)
Patch size		Broad		Existing vegetation (LANDFIRE 2014a)	Biophysical Setting (LANDFIRE 2010)
Structural connectivity		Broad		Existing vegetation (LANDFIRE 2014a)	Biophysical Setting (LANDFIRE 2010)
Development	Broad		Broad	(Carr et al. 2017)	Absence of development

Metrics measured by the BLM at the level of field sites (*n* = 2314 sites) include the composition of herbaceous vegetation, presence of plants of management concern, percent cover of bare ground and non-native invasive plant species, and percentage of area in large canopy gaps (≥200 cm, see Herrick et al. 2009 for methods). These data are publicly available at landscape.blm.gov. Of these metrics, we included cover of non-native invasive species as one of the six variables in our assessment. We focused on cover of non-native invasive species for three primary reasons. First, there are no readily available, quantitative natural reference or desired conditions for other site-level metrics. Second, cover of non-native invasive species is explicitly related to actions in Nevada land-use plans (e.g., BLM 2015a). Third, cover of non-native invasive plants, especially cheatgrass, strongly affects fire dynamics, with cascading effects on ecosystem structure, composition, and function.

In addition, we quantified five metrics at 30–90 m resolution from publicly available remotely sensed data. These metrics included the area and degree of alteration of shrublands, the size of shrubland patches, the proximity of shrubland pixels (a measure of shrubland connectivity), and an index of the surface footprint of terrestrial development from energy infrastructure, mineral extraction, agriculture, transportation, and urban development within a 2.5-km moving window (Carr et al. 2017). The BLM and stakeholders on their Resource Advisory Councils (BLM 2017b) have established vegetation amount, patch size, and connectivity as indicators of land health (Karl and Kachergis 2015). Degree of vegetation alteration has been used in other ecological integrity assessments for large areas (Walston and Hartmann 2018), and the area of surface development affects the composition and function of nearby natural areas (Forman and Alexander 1998;, Dale et al. 2005; Hansen et al. 2005; Leu et al. 2008; Jones et al. 2015).

### Implementing the Assessment

Our primary goal was to develop and illustrate a transferable process for assessing ecological integrity across large, multiple-use landscapes. Although BLM has identified standardized methods and metrics for monitoring vegetation at individual sites (Taylor et al. 2014), it has not yet developed standardized landscape-level indicators and methods (Wood et al. 2017). Therefore, our assessment incorporated landscape-level metrics that build on previous work (Wood et al. 2017) and can be quantified with data that are available across the western United States. We quantified current shrubland area by identifying those 30-m pixels for which the system group physiognomy in the Landscape Fire and Resource Management Planning Tools Project (LANDFIRE) existing vegetation type (EVT) layer was shrubland (LANDFIRE 2014a). We assessed shrubland alteration with the vegetation departure (VDEP) layer in LANDFIRE, which quantifies changes in species composition, structural stage, and canopy closure between estimated historical (pre-EuroAmerican settlement) and current vegetation (LANDFIRE 2014b). To define shrubland patches, we used the Region Group tool in ArcGIS version 10.3.1 (ESRI 2015), which identifies the region (here, patch) to which each cell belongs, and an eight-cell neighborhood rule (two or more pixels connected along a diagonal are considered to be a continuous patch). We used the Euclidean distance tool in ArcGIS version 10.3.1 (ESRI 2015) to calculate a simple metric of shrubland connectivity on the basis of distances between individual shrubland pixels. We conducted all spatial analyses in ArcGIS version 10.3.1 (ESRI 2015).

We defined natural reference conditions on the basis of publicly available quantitative data (Table 2). We used LANDFIRE biophysical settings (with the attribute GroupVeg domain value set to shrubland) to estimate reference conditions for shrubland area, vegetation departure, patch size, and shrubland connectivity. LANDFIRE biophysical settings represent the vegetation that may have been dominant prior to EuroAmerican settlement and are based on both the current biophysical environment and an approximation of the historical disturbance regime (LANDFIRE 2010). For simplicity and because, as noted above, we do not have information on the natural range of variability of our metrics (Table 2), we report departure from available reference conditions for each metric in five quantiles. Quantitative desired conditions have not yet been identified at the ecosystem level for these metrics in BLM resource management plans for Nevada (A. Titolo, pers. comm.).

**Fig. 5:**
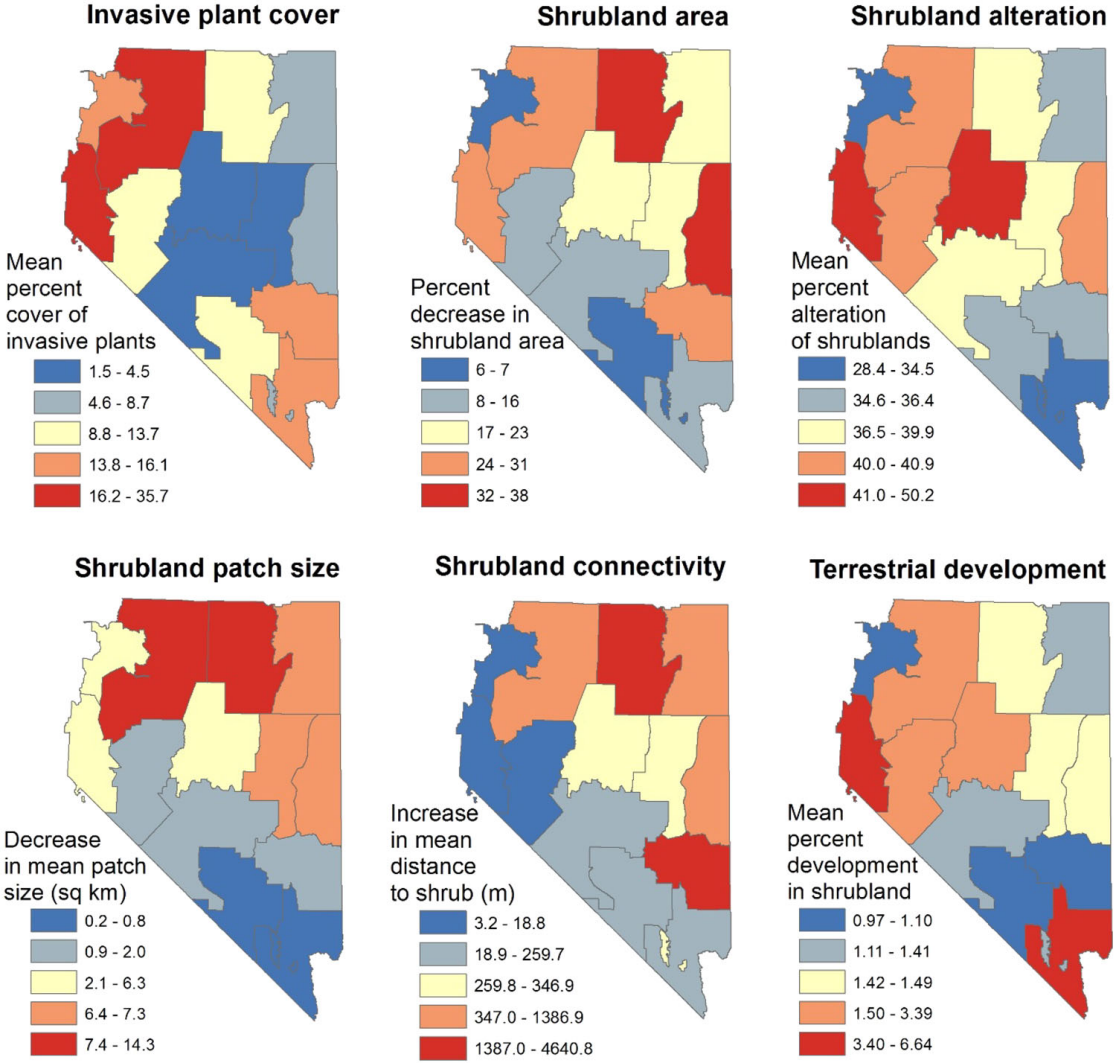
Ecological integrity of shrublands in Nevada summarized at the level of the 14 Bureau of Land Management (BLM) field offices contained entirely within Nevada. See Figs. 6 and 7 and the Supplementary Material for values of each metric

**Fig. 6:**
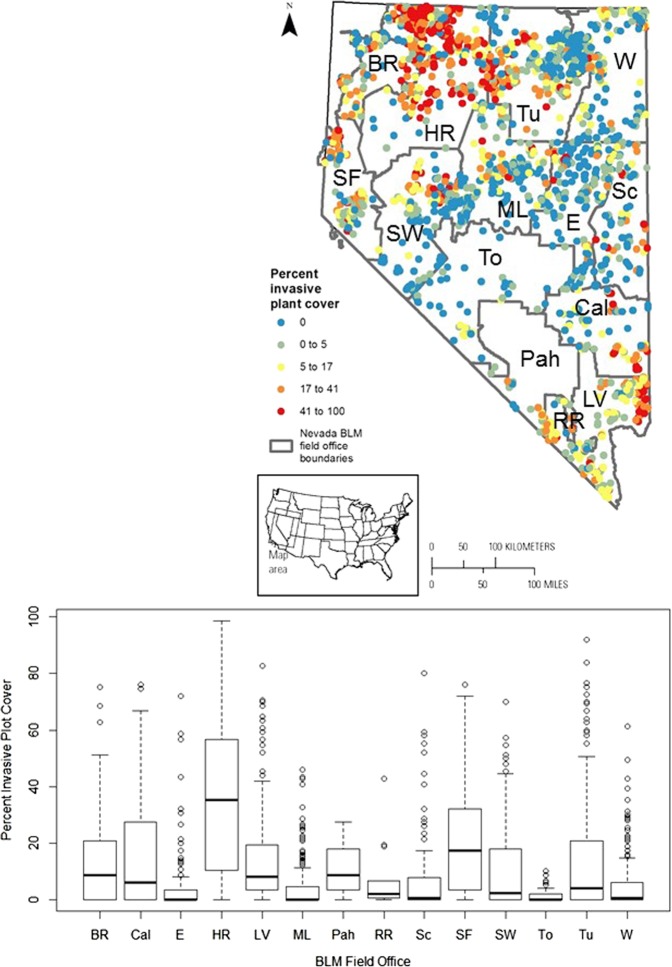
Cover of non-native invasive plant species in 14 Bureau of Land Management (BLM) field offices in Nevada. Field office names and abbreviations are as follows: Black Rock (BR), Caliente (Cal), Egan (E), Humboldt River (HR), Las Vegas (LV), Mount Lewis (ML), Pahrump (Pah), Red Rock/Sloan (RR), Schell (Sc), Sierra Front (SF), Stillwater (SW), Tonopah (To), Tuscarora (Tu), and Wells (W)

**Fig. 7:**
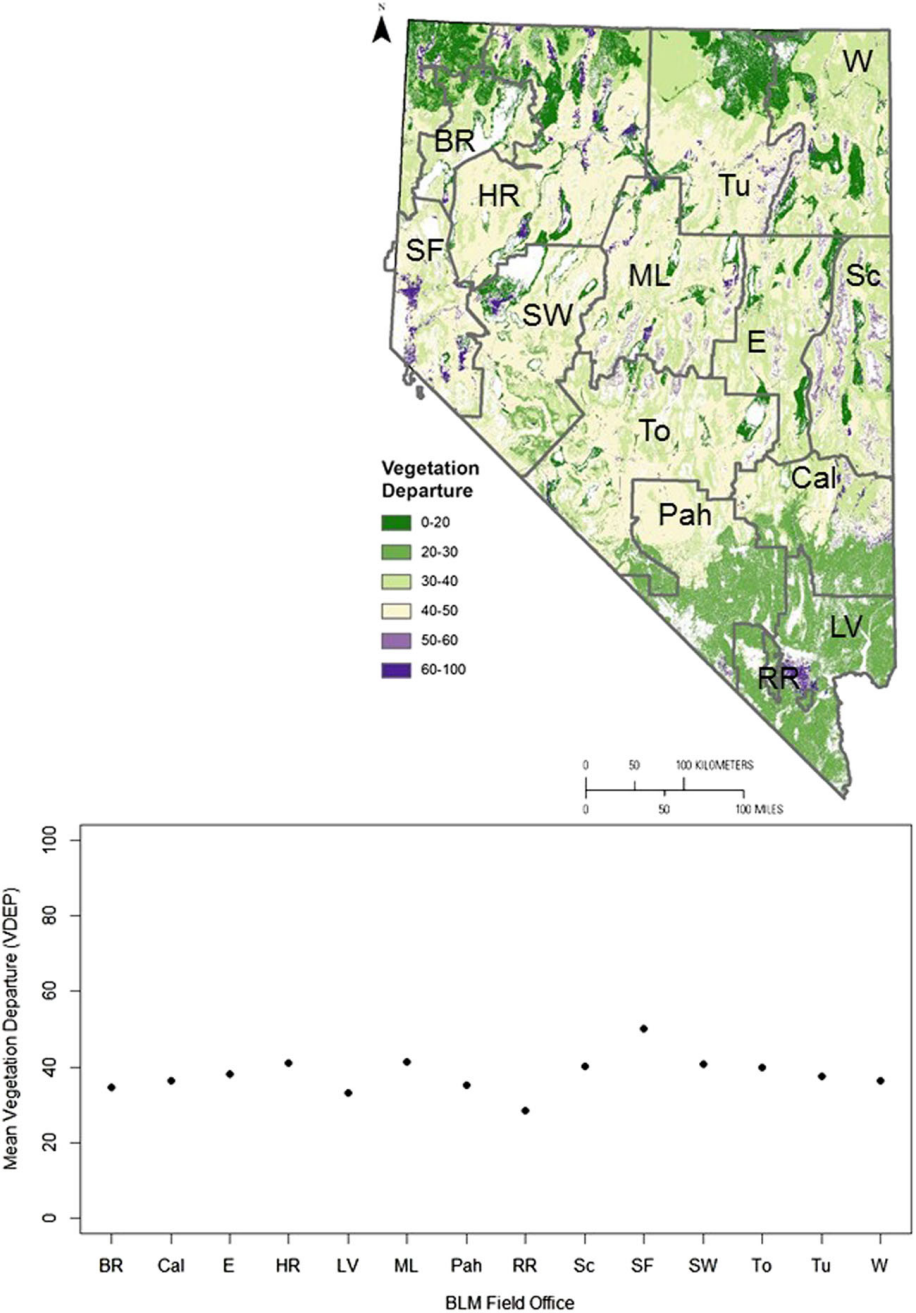
Alteration of shrublands in 14 Bureau of Land Management (BLM) field offices in Nevada. See Fig. 6 for the names and abbreviations of BLM field offices

We present our ecological integrity results at two spatial scales to inform two of BLM’s decision processes. The first decision, made by BLM state offices, is how to distribute funds for vegetation management and restoration projects among BLM field offices within the state (Fig. 5 can inform this decision). The second decision, made by BLM field offices, is what types and locations of vegetation management or restoration projects are of highest priority within their field office (Figs. 6 and 7 and Supplementary Figs. 1–4 can inform this decision).

Relative degree of departure from reference conditions varied among field office locations and ecological integrity metrics (Figs 5–7, Supplementary Figs 1–4), suggesting that different shrubland management strategies and actions will be most effective in different areas of the state. Cover of non-native invasive plants was lowest (1.5–4.5%) in three field districts in central Nevada (Figs. 5,6), but fire probability increases considerably even at these percentages (Bradley et al. 2018). Cover of non-native invasive plants can vary dramatically across relatively small areas (Fig. 6). Therefore, spatial data on cover of non-native invasive plants can inform strategies for their management (Lodge et al. 2006) and actions that meet the objective of “treat[ing] areas that contain cheatgrass and other invasive or noxious species to minimize competition and favor establishment of desired species.” [BLM 2015a]).

Shrubland area in two field offices in northern and east-central Nevada has decreased by more than a third compared with historic reference conditions (Fig. 5 and Supplementary Fig. 1). These results can inform decisions about where BLM concentrates sagebrush restoration efforts (e.g., “emphasiz[ing] treatment areas that have the best potential to maintain desired conditions or respond and return to the desired range of conditions and mosaic upon the landscape” [BLM 2008]). Shrubland alteration was greatest (41–50%) in two field offices in central and western Nevada (Fig. 5), with localized areas of very high alteration within those field offices (Fig. 7), indicating that it may be appropriate to evaluate the extent to which vegetation composition and structure in these areas are meeting the needs of Greater Sage-Grouse or other species of conservation concern, and what restoration methods might be most feasible and effective given their landscape context (e.g., the Sierra Front field office includes the large city of Reno). In northern Nevada, both patch size and shrubland proximity were much lower than reference conditions (Fig. 5 and Supplementary Figs. 2, 3), suggesting that restoration efforts might seek to increase the size and proximity of existing patches (Pyke et al. 2015).

Surface development within areas that historically were shrublands was most extensive (3.4–6.6%) in the field offices that encompass large cities (Reno and Las Vegas), but also exceeded 1.5% (second quintile) in three field offices in central and northwestern Nevada (Fig. 5 and Supplementary Fig. 4). Nevada resource management plans (e.g., BLM 2008) state that sagebrush systems should be managed for the benefit of pygmy rabbits (*Brachylagus idahoensis*) and Greater Sage-Grouse, and even low densities of development (e.g., 1–2% of the landscape) can negatively affect the presence, abundance, and habitat use of these two species (Kirol et al. 2015; Germaine et al. 2017). Nevada resource management plans also limit surface disturbance in some areas of shrubland (e.g., high-priority habitat for Greater Sage-Grouse, BLM 2015a).

## Discussion

Effective management of multiple-use lands requires openly acknowledging and regularly monitoring trade-offs between resource uses and resource conditions (Freeman et al. 2015; Seidl et al. 2016). Humans value nature and the services it provides (e.g., Costanza et al. 1997; Kotchen and Reiling 2000; Ojea and Loureiro 2007), and systems with greater integrity may provide more diverse services and greater levels of services (e.g., Balmford et al. 2002; Martin and Watson 2016). Pressure also is increasing to allow more intensive and extractive uses of public lands (USDOI 2017), and the BLM is required by law to assess whether these uses are degrading the health of the lands that it manages. As such, the BLM and other management agencies need practical approaches for conducting such assessments that are feasible to apply, easy to understand, and able to inform future management actions and permitting decisions.

BLM’s desire to explore the use of ecological integrity assessments began in 2010, when it started conducting ecoregional assessments across much of the western United States (BLM 2012). At that time, the concept of ecological integrity was not developed to the point that it could be applied to extensive, multiple-use landscapes (D. Wood, pers. obs.). Instead, most existing applications of terrestrial ecological integrity focused only on conservation (e.g., Parrish et al. 2003) and used wildlife species-based metrics (e.g., Glennon and Porter 2005), which limits applicability for a multiple-use agency that does not regularly monitor animals. The National Park Service has been instrumental in advancing the application of ecological integrity in the United States (e.g., Tierney et al. 2009; Mitchell et al. 2014), but their work focuses on discrete and relatively small land holdings typical of the National Park Service.

Given the challenges of implementing spatially extensive assessments, the U.S. Geological Survey and the BLM held a workshop in October 2014 to initiate development of methods for quantifying ecological integrity. Consistent with the principles of conducting science to inform and improve decisions (e.g., actionable science, translational ecology; Seavey and Howell 2010; Palmer 2012; Enquist et al. 2017), a group of scientists from multiple agencies and universities continued to meet periodically over the next 3 years to clearly define the term and develop a feasible method for applying such assessments.

The method we propose builds on experience from aquatic systems, addresses identified limitations of ecological integrity assessments, accommodates large landscapes in which jurisdictions are disjunct, and facilitates incorporation of assessment results into planning and management. The method is transparent and transferable, and incorporates four actions to facilitate its practicality for informing multiple-use resource management: use of existing agency policies, plans, and monitoring programs to inform metric selection; use of both resource- and stressor-based metrics; reliance on publicly available data collected at multiple scales; and comparison with both natural reference and societally desired conditions.

BLM is committed to providing multiple-scale scientific information to improve the transparency, consistency, and durability of its management decisions (Kitchell et al. 2015). Results of our case study assessment relate clearly to identified management objectives and strategies for non-native invasive species and sagebrush restoration in Nevada (e.g., BLM 2008, 2015a). However, under current Department of the Interior priorities, it is uncertain whether ecological integrity assessments will be applied and used to inform future BLM management actions. Tangible evidence of use would include conducting and applying ecological integrity assessments to identify quantitative resource objectives and associated management actions in future BLM resource management plans, and use of assessment results to evaluate plan effectiveness and to prioritize and target restoration and other management actions.

Our case study assessment highlights the variability observed across both space and metrics. No single field office had a consistent score across all metrics, and no field office had the same pattern of variability among metrics. Assessments based on larger numbers of metrics have yielded similar variability (Cleland et al. 2017), emphasizing the value of presenting results for each metric separately at a spatial resolution that is relevant for managers. Within BLM field offices, field data then can be used both to ground-truth results derived from remotely sensed metrics and to target individual restoration and management actions.

Our case study assessment was limited in multiple ways. We suggest that future assessments consider more refined metrics (e.g., a more comprehensive, distance-based metric of connectivity, Moilanen and Nieminen 2002; Simpkins et al. 2018) that reflect limiting factors of priority resources in the region. We also suggest exploring ways to represent uncertainty in metrics derived from remotely sensed landcover data, particularly for vegetation types that tend to occur in very dry or very wet areas, which often have lower classification accuracy (e.g., LANDFIRE 2015). Quantitative data to describe natural reference conditions (particularly field data) and societally desired conditions are also limited; in our case a lack of available reference conditions limited our analysis to 6 of the 11 proposed metrics. Ongoing sampling of privately owned grasslands and shrublands by the Natural Resources Conservation Service (USDA 2014), and recent sampling of public lands managed by the BLM across the western United States with comparable methods (Taylor et al. 2014), soon may provide a statistically robust number of samples in minimally disturbed grasslands and shrublands. In cases where quantitative desired conditions have not yet been identified in land-use plans, one may use ecological information on priority resources in the region (e.g., the degree of shrubland connectivity necessary to sustain populations of individual species) to help identify the point at which individual metrics may limit sustainability of the system or species. A greater focus on quantifying desired resource conditions, especially given projected changes in climate and land-use (Wurtzeback and Schultz 2016; Timberlake and Schultz 2017), is already evident within the BLM (e.g., BLM 2015a) and may facilitate integrity assessments that better can inform ecosystem management on public lands.

## Supplementary Information

Supplementary Information
