# Supplementary material for: Quantifying Ecological Integrity of Terrestrial Systems to Inform Management of Multiple-Use Public Lands in the United States
Source: Environ Manage. 2019 Apr 13;64(1):1–19. doi: 10.1007/s00267-019-01163-w (PMC6598959; doi:10.1007/s00267-019-01163-w)
Supplement: Supplementary file 1 — Supplementary Information [file 267_2019_1163_MOESM1_ESM.docx]

**Supplementary Material**

Fig. 1. Current and historical extent of shrublands in 14 Bureau of Land Management (BLM) field offices in Nevada. Field office names and abbreviations are in Fig 6.


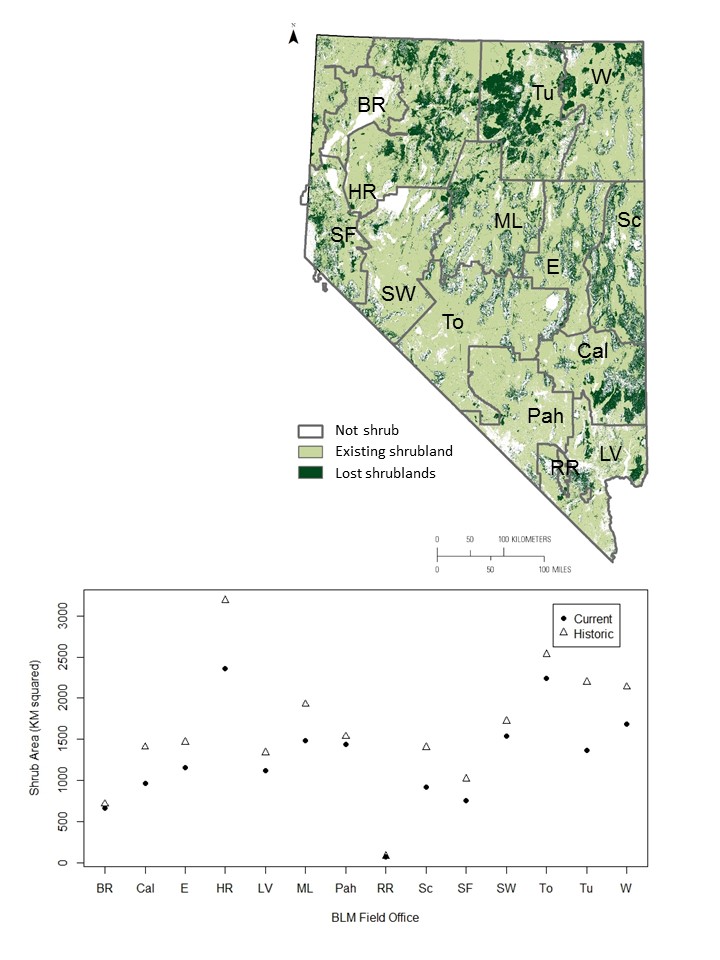


Fig. 2. Mean size of current and historic shrubland patches in 14 Bureau of Land Management (BLM) field offices in Nevada. Abbreviations for each BLM field office are in Fig. 6.
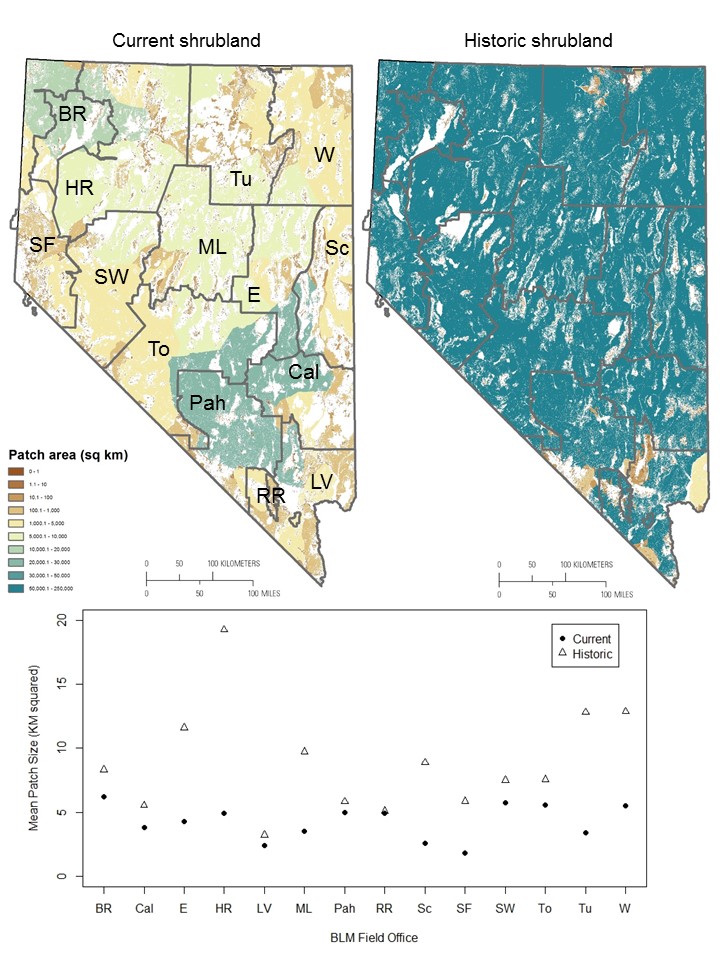


Fig. 3. Proximity of current and historic shrublands in 14 Bureau of Land Management (BLM) field offices in Nevada. Abbreviations for each BLM field office are in Fig. 6.


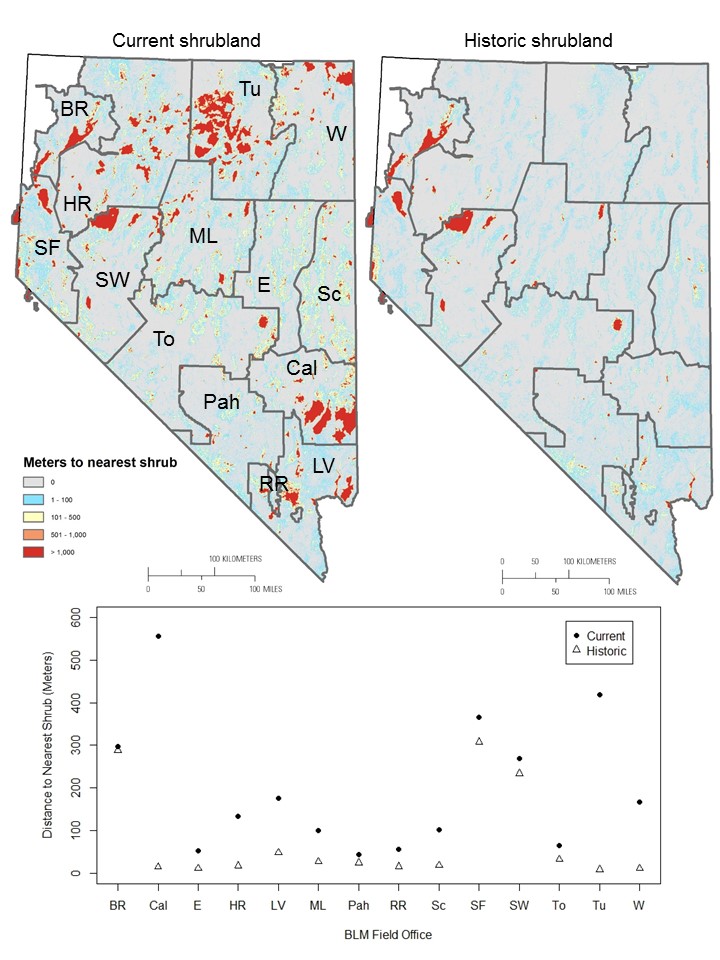


Fig. 4. Surface development (from energy infrastructure, mineral extraction, agriculture, transportation, and urban development) in 14 Bureau of Land Management (BLM) field offices in Nevada. Abbreviations for each BLM field office are in Fig. 6.**
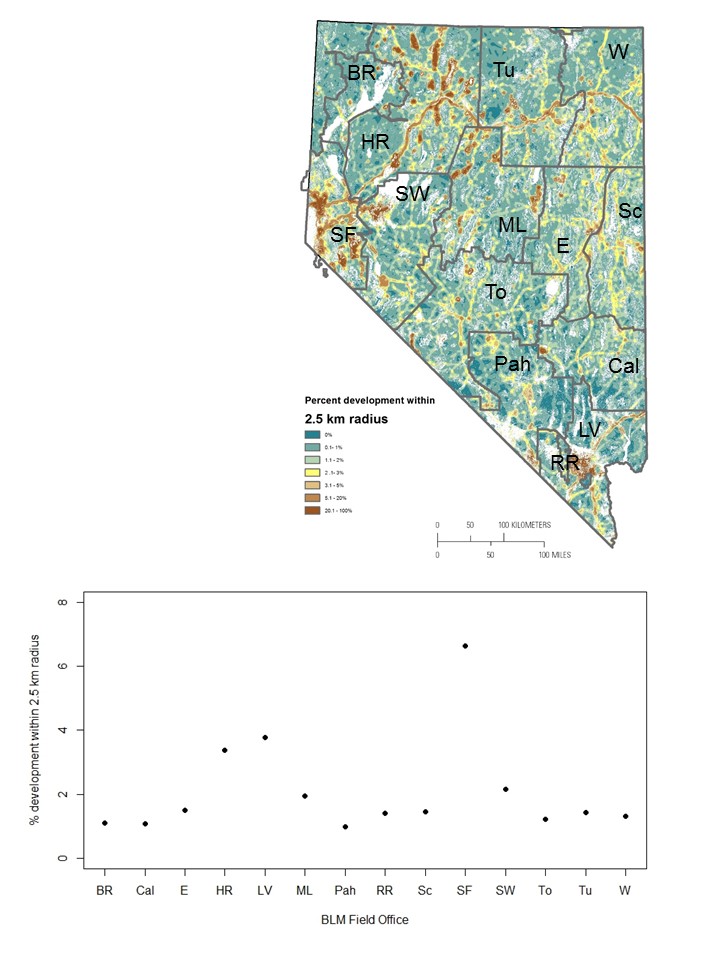
**
